# Guanidinium 2-phenyl­acetate

**DOI:** 10.1107/S1600536810025821

**Published:** 2010-07-07

**Authors:** Graham Smith, Urs D. Wermuth

**Affiliations:** a Faculty of Science and Technology, Queensland University of Technology, GPO Box 2434, Brisbane, Queensland 4001, Australia; b School of Biomolecular and Physical Sciences, Griffith University, Nathan, Queensland 4111, Australia

## Abstract

In the structure of the title salt, CH_6_N_3_^+^·C_8_H_7_O_2_^−^, the guanidinium cation gives three cyclic hydrogen-bonding inter­actions with O-atom acceptors of three independent phenyl­acetate anions, one *R*_2_^2^(8) and two *R*_2_^1^(6), giving one-dimensional columnar structures which extend down the 4_2_ axis in the tetra­gonal cell. Within these structures, there are solvent-accessible voids of volume 86.5 Å^3^.

## Related literature

For the structures of simple monocyclic aromatic guanidinium carboxyl­ates, see: Pereira Silva *et al.* (2007 ▶, 2010 ▶); Kleb *et al.* (1998 ▶); Smith & Wermuth (2010 ▶). For graph-set analysis, see: Etter *et al.* (1990 ▶). 
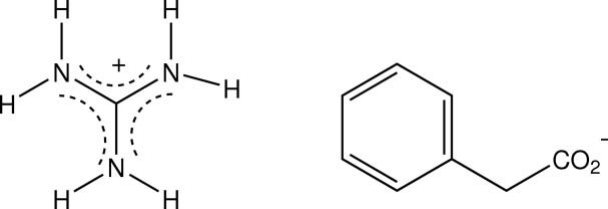


## Experimental

### 

#### Crystal data

CH_6_N_3_^+^·C_8_H_7_O_2_^−^
*M* *_r_* = 195.22
Tetragonal, 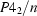

*a* = 16.8418 (10) Å
*c* = 7.8372 (6) Å
*V* = 2223.0 (3) Å^3^
*Z* = 8
Mo *K*α radiation
μ = 0.09 mm^−1^
*T* = 200 K
0.30 × 0.25 × 0.20 mm

#### Data collection

Oxford Diffraction Gemini-S CCD-detector diffractometer
7477 measured reflections
2191 independent reflections
1430 reflections with *I* > 2σ(*I*)
*R*_int_ = 0.027

#### Refinement

*R*[*F* ^2^ > 2σ(*F* ^2^)] = 0.040
*wR*(*F* ^2^) = 0.101
*S* = 0.93
2191 reflections
151 parameters
H atoms treated by a mixture of independent and constrained refinement
Δρ_max_ = 0.14 e Å^−3^
Δρ_min_ = −0.14 e Å^−3^

### 

Data collection: *CrysAlis PRO* (Oxford Diffraction, 2009 ▶); cell refinement: *CrysAlis PRO*; data reduction: *CrysAlis PRO*; program(s) used to solve structure: *SHELXS97* (Sheldrick, 2008 ▶); program(s) used to refine structure: *SHELXL97* (Sheldrick, 2008 ▶); molecular graphics: *PLATON* (Spek, 2009 ▶); software used to prepare material for publication: *PLATON*.

## Supplementary Material

Crystal structure: contains datablocks global, I. DOI: 10.1107/S1600536810025821/bv2147sup1.cif

Structure factors: contains datablocks I. DOI: 10.1107/S1600536810025821/bv2147Isup2.hkl

Additional supplementary materials:  crystallographic information; 3D view; checkCIF report

## Figures and Tables

**Table 1:** Hydrogen-bond geometry (Å, °)

*D*—H⋯*A*	*D*—H	H⋯*A*	*D*⋯*A*	*D*—H⋯*A*
N1*G*—H11*G*⋯O22^i^	0.86 (2)	2.02 (2)	2.876 (2)	173.9 (15)
N1*G*—H12*G*⋯O21	0.866 (16)	2.123 (17)	2.900 (2)	149.0 (15)
N2*G*—H21*G*⋯O22^ii^	0.834 (16)	2.219 (17)	2.9625 (19)	148.5 (15)
N2*G*—H22*G*⋯O21^i^	0.86 (2)	1.97 (2)	2.827 (2)	172.6 (15)
N3*G*—H31*G*⋯O21	0.897 (16)	2.068 (16)	2.8634 (17)	147.2 (13)
N3*G*—H32*G*⋯O22^ii^	0.859 (16)	2.073 (16)	2.8520 (17)	150.5 (15)

Symmetry codes: (i) 
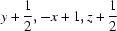
; (ii) 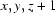.

**Fig. 1.**
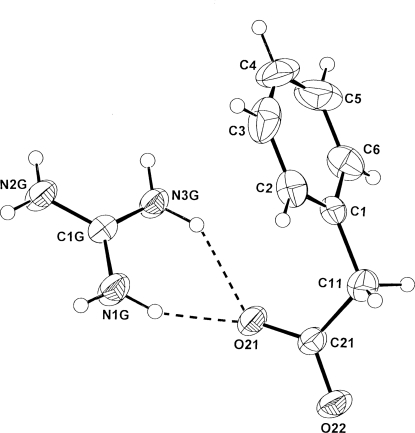
Molecular configuration and atom naming scheme for the guanidinium cation and the phenylacetate anion in (I). Inter-species hydrogen bonds are shown as dashed lines. Displacement ellipsoids are drawn at the 40% probability level.

**Fig. 2.**
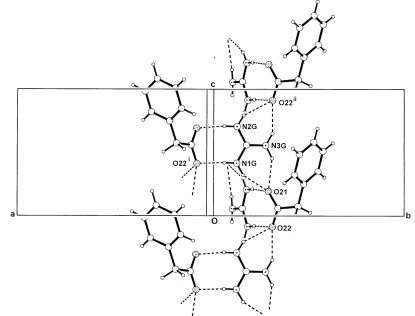
The hydrogen-bonding extensions of the basic asymmetric unit in the structure of (I), showing the three cyclic cation–anion hydrogen-bonding associations as dashed lines. Non-associative hydrogen atoms are deleted. For symmetry codes, see Table 1.

**Fig. 3.**
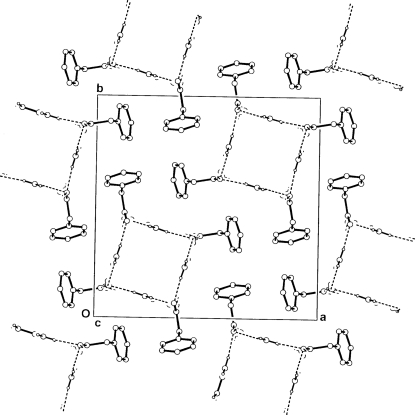
The hydrogen-bonded columnar structures of (I) viewed down the c axial direction of the tetragonal unit cell. Non-associative hydrogen atoms are deleted.

## supplementary crystallographic information

### Comment

The known structures of the guanidinium salts of simple monocyclic aromatic carboxylic acids comsist of those with benzoic acid (Pereira Silva *et al.*, 2007), 4-aminobenzoic acid (Pereira Silva *et al.*, 2010), 4-nitrobenzoic acid (Kleb *et al.*, 1998) and 3-nitrobenzoic acid (Smith & Wermuth, 2010). In these anhydrous structures the guanidinium cation is usually involved in cyclic hydrogen-bonding associations through *N*–*H*···O_carboxyl_ links [graph sets *R*_2_^2^(8) or *R*_2_^1^(6) (Etter *et al.*, 1990)] giving most commonly three-dimensional structures. The structure of the guanidinium salt of phenylacetic acid had not been previously reported so we carried out the 2:1 stoichiometric reaction of phenylacetic acid with guanidinium carbonate in aqueous ethanol solution, providing colourless crystals of the title compound, CH_6_N_3_^+^ C_8_H_7_O_2_^-^ (I) when recrystallized from water.

In the structure of (I) (Figs. 1, 2), each guanidinium cation is involved in three cyclic hydrogen-bonding interactions with the carboxyl O-acceptors of three independent phenylacetate anions, one *R*_2_^2^(8) and two *R*_2_^1^(6). These result in un-associated one-dimensional columnar structures which extend down the 4_2_ (*c*) axis in the tetragonal cell (Fig. 3). Within these columnar structures there are 86.5 Å^3^ solvent accessible voids which are large enough to accommodate water molecules but surprisingly do not, despite the sample having been obtained by recrystallization from water.

With the anion, the acetate substituent is close to normal to the plane of the benzene ring [torsion angle C2–C1–C11–C21, 86.98 (18)°]. Present in the benzene ring are unexplained high unidirectional displacement parameters for three atoms [C3, C4, C5: *U*_11_, 0.1009 (18), 0.185 (3), 0.1019 (18) Å^2^ respectively, *cf.* a typical value 0.0427 (9) Å^2^ for C2].

### Experimental

The title compound was synthesized by heating together under reflux for 10 minutes 1 mmol of phenylacetic acid and 0.5 mmol of guanidinium carbonate in 50 ml of 50% ethanol-water. After concentration to *ca* 30 ml, room temperature evaporation of the hot-filtered solution gave a colourless powder which was recrystallized from a minimum volume of water, giving on total evaporation, crystal plates of (I) (m.p. 443 K), from which a specimen suitable for X-ray analysis was cleaved.

### Refinement

Hydrogen atoms involved in hydrogen-bonding interactions were located by difference methods and their positional and isotropic displacement parameters were refined. The H atoms were included in the refinement in calculated positions (C–H_aromatic_ = 0.93 Å and C–H_aliphatc_ = 0.97 Å) and treated as riding, with *U*_iso_(H) = 1.2*U*_eq_(C).

### Crystal data

**Table:**

CH_6_N_3_^+^·C_8_H_7_O_2_^−^	*D*_x_ = 1.167 Mg m^−^^3^
*M**_r_* = 195.22	Melting point: 443 K
Tetragonal, *P*4_2_/*n*	Mo *K*α radiation, λ = 0.71073 Å
Hall symbol: -P 4bc	Cell parameters from 2510 reflections
*a* = 16.8418 (10) Å	θ = 3.1–28.6°
*c* = 7.8372 (6) Å	µ = 0.09 mm^−^^1^
*V* = 2223.0 (3) Å^3^	*T* = 200 K
*Z* = 8	Block, colourless
*F*(000) = 832	0.30 × 0.25 × 0.20 mm

### Data collection

**Table:**

Oxford Diffraction Gemini-S CCD-detector diffractometer	1430 reflections with *I* > 2σ(*I*)
Radiation source: Enhance (Mo) X-ray source	*R*_int_ = 0.027
graphite	θ_max_ = 26.0°, θ_min_ = 3.1°
ω scans	*h* = −20→18
7477 measured reflections	*k* = −10→20
2191 independent reflections	*l* = −9→8

### Refinement

**Table:**

Refinement on *F*^2^	Primary atom site location: structure-invariant direct methods
Least-squares matrix: full	Secondary atom site location: difference Fourier map
*R*[*F*^2^ > 2σ(*F*^2^)] = 0.040	Hydrogen site location: inferred from neighbouring sites
*wR*(*F*^2^) = 0.101	H atoms treated by a mixture of independent and constrained refinement
*S* = 0.93	*w* = 1/[σ^2^(*F*_o_^2^) + (0.0592*P*)^2^] where *P* = (*F*_o_^2^ + 2*F*_c_^2^)/3
2191 reflections	(Δ/σ)_max_ = 0.001
151 parameters	Δρ_max_ = 0.14 e Å^−^^3^
0 restraints	Δρ_min_ = −0.14 e Å^−^^3^

### Special details

**Table:**

Geometry. Bond distances, angles *etc*. have been calculated using the rounded fractional coordinates. All su's are estimated from the variances of the (full) variance-covariance matrix. The cell e.s.d.'s are taken into account in the estimation of distances, angles and torsion angles
Refinement. Refinement of *F*^2^ against ALL reflections. The weighted *R*-factor *wR* and goodness of fit *S* are based on *F*^2^, conventional *R*-factors *R* are based on *F*, with *F* set to zero for negative *F*^2^. The threshold expression of *F*^2^ > σ(*F*^2^) is used only for calculating *R*-factors(gt) *etc*. and is not relevant to the choice of reflections for refinement. *R*-factors based on *F*^2^ are statistically about twice as large as those based on *F*, and *R*- factors based on ALL data will be even larger.

### Fractional atomic coordinates and isotropic or equivalent isotropic displacement parameters (Å^2^)

**Table:**

	*x*	*y*	*z*	*U*_iso_*/*U*_eq_	
O21	0.64098 (7)	0.43351 (6)	0.19316 (12)	0.0534 (4)	
O22	0.62981 (7)	0.44279 (6)	−0.08684 (12)	0.0518 (4)	
C1	0.61757 (8)	0.59370 (8)	0.25158 (18)	0.0364 (5)	
C2	0.68820 (9)	0.61788 (9)	0.3219 (2)	0.0510 (6)	
C3	0.69086 (15)	0.64720 (11)	0.4853 (3)	0.0775 (9)	
C4	0.6222 (2)	0.65222 (12)	0.5811 (2)	0.0918 (11)	
C5	0.55179 (15)	0.62730 (13)	0.5105 (3)	0.0817 (9)	
C6	0.54971 (10)	0.59874 (10)	0.3487 (2)	0.0554 (6)	
C11	0.61473 (11)	0.56093 (9)	0.07399 (18)	0.0550 (6)	
C21	0.62985 (8)	0.47232 (9)	0.05979 (17)	0.0378 (5)	
N1G	0.77589 (11)	0.40624 (9)	0.41545 (18)	0.0537 (5)	
N2G	0.77186 (10)	0.40368 (9)	0.70667 (17)	0.0517 (5)	
N3G	0.66128 (8)	0.43990 (8)	0.55565 (18)	0.0445 (5)	
C1G	0.73652 (9)	0.41692 (8)	0.55946 (17)	0.0381 (5)	
H2	0.73470	0.61440	0.25830	0.0610*	
H3	0.73900	0.66370	0.53150	0.0930*	
H4	0.62360	0.67210	0.69180	0.1100*	
H5	0.50520	0.63000	0.57400	0.0980*	
H6	0.50160	0.58230	0.30260	0.0670*	
H11	0.65390	0.58860	0.00530	0.0660*	
H12	0.56290	0.57230	0.02590	0.0660*	
H11G	0.8248 (12)	0.3924 (10)	0.4193 (18)	0.054 (5)*	
H12G	0.7504 (10)	0.4141 (10)	0.321 (2)	0.063 (5)*	
H21G	0.7451 (10)	0.4104 (9)	0.795 (2)	0.049 (5)*	
H22G	0.8201 (12)	0.3872 (10)	0.710 (2)	0.061 (6)*	
H31G	0.6432 (9)	0.4549 (9)	0.453 (2)	0.049 (5)*	
H32G	0.6410 (10)	0.4547 (10)	0.651 (2)	0.055 (5)*	

### Atomic displacement parameters (Å^2^)

**Table:**

	*U*^11^	*U*^22^	*U*^33^	*U*^12^	*U*^13^	*U*^23^
O21	0.0885 (9)	0.0400 (6)	0.0317 (6)	0.0054 (6)	−0.0136 (5)	−0.0003 (5)
O22	0.0728 (8)	0.0548 (7)	0.0279 (6)	−0.0017 (6)	0.0036 (5)	−0.0026 (5)
C1	0.0399 (8)	0.0297 (8)	0.0397 (8)	0.0033 (7)	−0.0008 (7)	0.0028 (6)
C2	0.0427 (9)	0.0458 (10)	0.0646 (11)	−0.0026 (8)	−0.0051 (8)	0.0073 (8)
C3	0.1009 (18)	0.0509 (12)	0.0807 (15)	−0.0161 (12)	−0.0466 (13)	0.0084 (11)
C4	0.185 (3)	0.0493 (12)	0.0411 (11)	0.0059 (15)	−0.0096 (14)	−0.0145 (9)
C5	0.1019 (18)	0.0733 (14)	0.0699 (14)	0.0135 (13)	0.0366 (13)	−0.0137 (12)
C6	0.0406 (10)	0.0558 (11)	0.0698 (12)	0.0006 (8)	0.0068 (8)	−0.0051 (9)
C11	0.0797 (13)	0.0446 (9)	0.0406 (9)	0.0064 (9)	−0.0023 (8)	0.0044 (7)
C21	0.0400 (8)	0.0445 (9)	0.0288 (8)	0.0002 (7)	0.0003 (6)	0.0004 (7)
N1G	0.0462 (9)	0.0832 (11)	0.0318 (8)	0.0094 (8)	0.0017 (7)	0.0011 (7)
N2G	0.0433 (9)	0.0805 (11)	0.0312 (8)	0.0052 (8)	−0.0022 (7)	−0.0033 (7)
N3G	0.0449 (8)	0.0599 (9)	0.0288 (8)	0.0051 (6)	0.0005 (6)	0.0030 (6)
C1G	0.0415 (9)	0.0413 (8)	0.0314 (8)	−0.0044 (7)	−0.0011 (7)	−0.0003 (6)

### Geometric parameters (Å, °)

**Table:**

O21—C21	1.2470 (17)	C1—C2	1.373 (2)
O22—C21	1.2522 (17)	C2—C3	1.373 (3)
N1G—C1G	1.321 (2)	C3—C4	1.381 (4)
N2G—C1G	1.317 (2)	C4—C5	1.374 (4)
N3G—C1G	1.325 (2)	C5—C6	1.357 (3)
N1G—H11G	0.86 (2)	C11—C21	1.518 (2)
N1G—H12G	0.866 (16)	C2—H2	0.9300
N2G—H21G	0.834 (16)	C3—H3	0.9300
N2G—H22G	0.86 (2)	C4—H4	0.9300
N3G—H31G	0.897 (16)	C5—H5	0.9300
N3G—H32G	0.859 (16)	C6—H6	0.9300
C1—C6	1.376 (2)	C11—H12	0.9700
C1—C11	1.498 (2)	C11—H11	0.9700
			
C1G—N1G—H12G	117.4 (11)	O21—C21—O22	124.14 (14)
H11G—N1G—H12G	123.3 (15)	C1—C2—H2	120.00
C1G—N1G—H11G	119.3 (10)	C3—C2—H2	120.00
C1G—N2G—H22G	120.6 (11)	C2—C3—H3	120.00
H21G—N2G—H22G	122.0 (15)	C4—C3—H3	120.00
C1G—N2G—H21G	117.4 (11)	C5—C4—H4	120.00
C1G—N3G—H32G	116.5 (11)	C3—C4—H4	121.00
H31G—N3G—H32G	124.3 (15)	C4—C5—H5	120.00
C1G—N3G—H31G	115.3 (10)	C6—C5—H5	120.00
C2—C1—C6	118.65 (14)	C1—C6—H6	119.00
C2—C1—C11	120.66 (13)	C5—C6—H6	119.00
C6—C1—C11	120.68 (14)	C21—C11—H11	109.00
C1—C2—C3	120.62 (16)	C21—C11—H12	108.00
C2—C3—C4	120.1 (2)	H11—C11—H12	108.00
C3—C4—C5	119.00 (18)	C1—C11—H11	108.00
C4—C5—C6	120.5 (2)	C1—C11—H12	108.00
C1—C6—C5	121.17 (17)	N2G—C1G—N3G	120.08 (14)
C1—C11—C21	115.15 (12)	N1G—C1G—N2G	119.89 (15)
O21—C21—C11	118.63 (12)	N1G—C1G—N3G	120.02 (14)
O22—C21—C11	117.24 (12)		
			
C6—C1—C2—C3	−0.6 (2)	C1—C2—C3—C4	0.4 (3)
C11—C1—C2—C3	−179.37 (15)	C2—C3—C4—C5	0.2 (3)
C2—C1—C6—C5	0.3 (2)	C3—C4—C5—C6	−0.5 (3)
C11—C1—C6—C5	179.08 (16)	C4—C5—C6—C1	0.2 (3)
C2—C1—C11—C21	86.98 (18)	C1—C11—C21—O21	2.0 (2)
C6—C1—C11—C21	−91.75 (18)	C1—C11—C21—O22	−178.42 (13)

### Hydrogen-bond geometry (Å, °)

**Table:**

*D*—H···*A*	*D*—H	H···*A*	*D*···*A*	*D*—H···*A*
N1G—H11G···O22^i^	0.86 (2)	2.02 (2)	2.876 (2)	173.9 (15)
N1G—H12G···O21	0.866 (16)	2.123 (17)	2.900 (2)	149.0 (15)
N2G—H21G···O22^ii^	0.834 (16)	2.219 (17)	2.9625 (19)	148.5 (15)
N2G—H22G···O21^i^	0.86 (2)	1.97 (2)	2.827 (2)	172.6 (15)
N3G—H31G···O21	0.897 (16)	2.068 (16)	2.8634 (17)	147.2 (13)
N3G—H32G···O22^ii^	0.859 (16)	2.073 (16)	2.8520 (17)	150.5 (15)

Symmetry codes: (i) *y*+1/2, −*x*+1, *z*+1/2; (ii) *x*, *y*, *z*+1.
